# Scarless deletion of up to seven methyl-accepting chemotaxis genes with an optimized method highlights key function of CheM in *Salmonella* Typhimurium

**DOI:** 10.1371/journal.pone.0172630

**Published:** 2017-02-17

**Authors:** Stefanie Hoffmann, Christiane Schmidt, Steffi Walter, Jennifer K. Bender, Roman G. Gerlach

**Affiliations:** 1 Project Group 5, Robert Koch Institute, Wernigerode, Germany; 2 Division of Nosocomial Pathogens and Antibiotic Resistances, Department of Infectious Diseases, Robert Koch Institute, Wernigerode, Germany; New York State Department of Health, UNITED STATES

## Abstract

Site-directed scarless mutagenesis is an essential tool of modern pathogenesis research. We describe an optimized two-step protocol for genome editing in *Salmonella enterica* serovar Typhimurium to enable multiple sequential mutagenesis steps in a single strain. The system is based on the λ Red recombinase-catalyzed integration of a selectable antibiotics resistance marker followed by replacement of this cassette. Markerless mutants are selected by expressing the meganuclease I-SceI which induces double-strand breaks in bacteria still harboring the resistance locus. Our new dual-functional plasmid pWRG730 allows for heat-inducible expression of the λ Red recombinase and tet-inducible production of I-SceI. Methyl-accepting chemotaxis proteins (MCP) are transmembrane chemoreceptors for a vast set of environmental signals including amino acids, sugars, ions and oxygen. Based on the sensory input of MCPs, chemotaxis is a key component for *Salmonella* virulence. To determine the contribution of individual MCPs we sequentially deleted seven MCP genes. The individual mutations were validated by PCR and genetic integrity of the final seven MCP mutant WRG279 was confirmed by whole genome sequencing. The successive MCP mutants were functionally tested in a HeLa cell infection model which revealed increased invasion rates for non-chemotactic mutants and strains lacking the MCP CheM (Tar). The phenotype of WRG279 was reversed with plasmid-based expression of CheM. The complemented WRG279 mutant showed also partially restored chemotaxis in swarming assays on semi-solid agar. Our optimized scarless deletion protocol enables efficient and precise manipulation of the *Salmonella* genome. As demonstrated with whole genome sequencing, multiple subsequent mutagenesis steps can be realized without the introduction of unwanted mutations. The sequential deletion of seven MCP genes revealed a significant role of CheM for the interaction of *S*. Typhimurium with host cells which might give new insights into mechanisms of *Salmonella* host cell sensing.

## Introduction

The ability for precise manipulation of bacterial genomes is of utmost importance in modern microbiological research. Although there is a long history of manipulating bacterial genomes, the application of phage-derived recombinases constitute a breakthrough in bacterial genetics [1, 2]. These “recombineering” strategies jointly exploit the ability of phage Rac RecE/T or of phage λ Red recombinases to use DNA fragments of less than 40 bp as substrates for homologous recombination [3]. Direct integration of these homologous sequences within short oligonucleotides is a great advantage which makes cloning of helper plasmids obsolete.

Over the years a vast number of different strategies and ever more refined protocols have been developed with a clear trend towards “scarless” genome manipulations [4–9]. These techniques are usually based on two steps starting with the integration of a selectable marker (e.g. an antibiotic resistance gene) followed by seamless replacement of that particular marker. For the second step efficient methods to select for loss of the marker are required. Here, accumulation of toxic metabolites based on *tetAR* [6, 10], sucrose sensitivity utilizing the *sacB* gene [7, 9], *rpsL*-mediated streptomycin sensitivity in resistant hosts [11, 12], the CcdA/CcdB toxin-antitoxin system [13] or I-SceI induced double-strand breaks (DSB) [4, 5, 14–17] were successfully used. The meganuclease I-SceI of *S*. *cerevisiae* has an unusually long recognition site of 18 bp which is statistically not present in bacterial genomes [18]. Mechanistically, the I-SceI site is co-integrated into the genome with the antibiotic resistance cassette during the first recombination step. Expression of the I-SceI enzyme after the second step selects for successful recombinants. Thus the precise and independent regulated expression of λ Red recombinase and I-SceI is a prerequisite for maximum efficiency and reliability of this method. Whereas the above mentioned methods rely on double-stranded DNA (dsDNA) as substrate for recombination, the chromosomal integration of short single-stranded DNA (ssDNA) oligonucleotides has also been demonstrated [3]. Recombineering of ssDNA requires only the function of λ Beta/RecT ssDNA binding proteins and functions without selectable or counter-selectable markers [19, 20]. However, methyl-directed mismatch repair reduces efficiency of the method and thereby increasing the effort of screening for correct clones [21].

Recombineering techniques are not only limited by the efficiency of the applied systems but also in general by the amenability of microorganisms to the Red/Rec recombinases. Alternative approaches are explored not only to avoid this limitation but to make genome editing even more efficient. The CRISPR/Cas9 (clustered regularly interspaced short palindromic repeats and its associated protein, Cas9) system promises many advantages and is tremendously successful for editing eukaryotic genomes. Unfortunately, its application in bacteria is still limited. Amongst other reasons this is mainly due to the lack of a non-homologous end joining mechanism for DNA repair in most bacteria (reviewed in [22]). Nevertheless, successful CRISPR/Cas9-mediated genome editing including the introduction of deletions, insertions, and point mutations, was demonstrated when combined with λ Red recombinase functions [23]. The system was especially promoted for introducing multiple genome modifications since it does not rely on the cyclic integration and excision of a selectable marker [24]. However, the method requires careful design of the specific sequences for the gene-targeting protospacer adjacent motif (PAM) in order to prevent potential off-target activity. In addition, the PAM sequence together with the single guide RNA has to be supplied on a plasmid which needs to be cloned and, ideally, sequence-verified for each target gene to achieve high efficiency in bacteria [8, 23, 24]. Until further optimization of CRISPR/Cas9 system for bacteria, refined scarless recombineering protocols are an efficient and cost-effective tool for genome editing. Based on our previously developed method [4] we optimized the system to enhance efficiency and enable fast and reliable sequential modifications of the *Salmonella* Typhimurium (STM) genome.

We wanted to demonstrate the functionality of the optimized protocol in STM deleting seven genes encoding for methyl-accepting chemotaxis proteins (MCPs). MCPs are sensor molecules which respond to a variety of environmental cues including amino acids, sugars, ions and oxygen. Receptor signaling is initiated by reversible ligand binding at the periplasmic domains of dimeric MCPs [25]. Upon activation the receptor-bound kinase CheA phosphorylates the response regulator CheY. Through binding to the flagellar motor complex CheY influences the direction of flagellar rotation. As a result environmental signals perceived by MCPs control chemotactic swimming (reviewed in [26]). *Salmonella* expresses homologs to the *E*. *coli* MCPs Tsr, Tar, Trg and Aer. Tsr and Tar detect the amino acids serine and aspartate, respectively [27, 28]. Although the *Salmonella* Tar homolog CheM shows only 79% sequence identity with Tar of *E*. *coli* it was also demonstrated to bind and respond to aspartate [29, 30]. Trg is responsible for the sensing of the sugars glucose, galactose and ribose [31]. Alterations of the redox potential can be detected by Aer [32]. In contrast to the above mentioned MCPs the chemotaxis sensors Tap, Tip, McpA, McpB and McpC are only found in *Salmonella*. Tap was shown to sense citrate and phenol [33] whereas McpB/C mediate a repellent response towards L-cystine [34]. Currently no function for McpA [35], lacking a transmembrane domain, and Tip which is devoid of the periplasmic sensor domain is known.

We used a thoroughly defined set of MCP mutants to investigate the impact of individual MCPs on the ability of *Salmonella* to invade HeLa cells. Our infection experiments revealed an increased invasion rate for mutants lacking CheM indicating a detrimental effect of CheM-mediated chemotaxis in this infection model.

## Results and discussion

### Design and construction of system components

**Fig 1:**
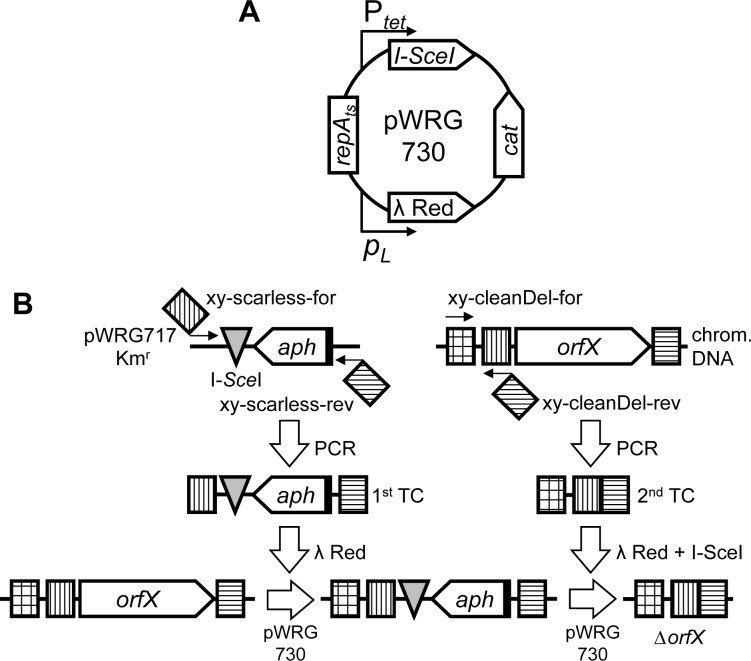
Overview of the method. (A) Schematic representation of the functional units of plasmid pWRG730. The operon containing λ Red recombinase functions is under control of the heat-inducible phage-derived promoter *p*_*L*_. Expression of the I-SceI meganuclease is controlled by a tetracycline-inducible promoter (P_*tet*_). A chloramphenicol resistance cassette (*cat*) is used for selection purposes. Due to its temperature-sensitive origin of replication (*repA*_*ts*_) the plasmid can be easily cured at elevated growth temperatures. (B) Representation of the two-step scarless deletion methodology. A kanamycin resistance cassette (*aph*) is amplified together with an I-SceI cleavage site (grey triangle) from pWRG717 with two 60-mer primers each containing site-specific homology extensions at their 5’-ends (striped squares). Chromosomal integration of this first targeting construct (TC) is achieved by λ Red recombinase expression from pWRG730. The 2^nd^ TC is also generated by PCR using chromosomal DNA as template and contains a direct fusion of up- and downstream homology regions. After genomic integration of the 2^nd^ TC using λ Red recombinase, successful recombinants are selected by I-SceI expression from pWRG730. A detailed description of the method can be found in the main text.

In our previously published system [4] the arabinose-inducible expression of the λ Red recombinase was combined with the meganuclease I-SceI under control of the *tetA* promoter in plasmid pWRG99 [36]. Although the system allowed for efficient generation of scarless deletions or single nucleotide exchanges, it required curing and re-transformation of pWRG99 between each recombination step [4]. We speculated that leaky expression of the λ Red proteins from the P_BAD_ promoter in the absence of glucose [37] could have selected for inactive recombinases. Therefore we reasoned that a differently regulated λ Red expression plasmid might circumvent this problem. The pSC101-based pSIM5 harbors the phage λ *p*_*L*_ operon comprising the genes *exo*, *bet* and *gam* under the control of the temperature-sensitive repressor CI857 that enables heat-inducible expression of λ Red recombinase functions [38]. Moreover, the pSIM5 plasmid allows for simple curing by its temperature-sensitive *repA*_*ts*_ origin of replication. From the functional perspective pSIM5 exhibited 10- to 60-fold higher recombination efficiency compared to the arabinose-inducible λ Red expression plasmid pKD119, which is similar to pKD46 except for the tetracycline resistance gene [38]. The combined λ Red/I-SceI expression plasmid pWRG730 was constructed by integrating the tetracycline-inducible I-SceI expression cassette of pWRG99 into the highly efficient pSIM5 vector (Fig 1A).

Together with pWRG730 new FRT-free template plasmids were constructed based on pBluescript II SK+. Flp recombinase target (FRT) sites as present on the old template plasmid pWRG100 negatively interfered with DSB-based counterselection [4]. To circumvent this problem, the kanamycin resistance cassette of pKD4 [36] was integrated in two orientations relative to the I-SceI cleavage site thereby producing pWRG717 (Fig 1B) and pWRG832 (not shown). To provide an alternative selection marker, a spectinomycin resistance cassette originating from pDL1098 [39] was cloned in both orientations which resulted in pWRG829 and pWRG865 (data not shown). Although all four template vectors are suitable for the introduction of deletions, heterologous DNA or nucleotide exchanges, we routinely used the template vectors with the resistance gene in reversed orientation compared to the I-SceI cleavage site (pWRG717, pWRG829) (Fig 1B and not shown) to minimize polar effects in the first recombination step. The vectors with the resistance cassette in same orientation to the I-SceI cleavage site enable the simple integration of the antibiotic resistance gene in an artificial operon structure to serve as a reporter gene as described for *Vibrio cholerae* [39].

### Sequential deletion of MCP genes

To evaluate the functionality of the system we decided to sequentially delete all genes encoding for known MCPs in *S*. Typhimurium strain NCTC 12023. The MCP genes were deleted in the following order: *aer*, *tcp*, *tsr*, *trg*, *cheM* (*tar*), *mcpC* (STM14_3893) and *mcpB* (STM14_3817) [35]. Each scarless deletion involved two successive recombination steps: (i) integration of a kanamycin resistance cassette amplified from pWRG717 and (ii) replacement of that resistance cassette by a PCR fragment containing the fused flanking regions of the gene to be deleted (Fig 1B) [4]. Similar strategies have been described before but with Red recombinase and I-SceI functionality provided on different plasmids [5] or requiring multiple rounds of culturing in selective medium for maximum efficiency [17]. In contrast to our first approach [4] the new plasmid pWRG730 encoding the recombinase and I-SceI meganuclease could be maintained within the bacteria during the whole sequential gene deletion process through selection with chloramphenicol (Cm) and keeping the cells at 30°C.

Primers ‘xy-scarless-for’ and ‘xy-scarless-rev’ were used for amplification of an I-SceI cleavage site together with a kanamycin resistance cassette from template vector pWRG717 to produce the 1^st^ targeting construct (TC) for recombination. Whereas the 20 bases at the 3’ ends of the primers were designed to bind to the template vectors, the 40 bases at each 5’ end are homologous to regions up- and downstream of ‘*orfX*’ and thus determine the genome integration site (Fig 1B). Electrocompetent *Salmonella* with heat-induced λ Red recombinase were transformed with the 1^st^ TC and transformants were selected on kanamycin (Km) and Cm-containing LB agar plates. Proper integration of the I-SceI/kanamycin cassette and replacement of each MCP gene was verified in both directions by PCR. The TC for the second recombination step was also generated by PCR. The construct represents a fusion of upstream and downstream homologous regions of the target deletion site. The homologous upstream sequence was amplified from wild-type (WT) chromosomal DNA as template using a short upstream-binding ‘xy-cleanDel-for’ primer and a 60-mer ‘xy-cleanDel-rev’ primer. The downstream homology region comprises the 40 bases of the 5’ end of ‘xy-cleanDel-rev’ (Fig 1B). This PCR-based approach to obtain a 2^nd^ TC is much more flexible and cost-effective for introducing deletions or for site-directed mutagenesis compared to phosphorylated oligonucleotides [4]. PCR using 60-mer primers with 40 bases homology extensions would be also the means of choice for insertion of heterologous DNA sequences. Alternatively, synthetic DNA with compatible terminal homology regions can be used in the 2^nd^ recombination step hence providing maximal flexibility. This step was selected on anhydrotetracycline (AHT) containing LB agar plates which induced I-SceI expression from pWRG730 allowing only the growth of successful recombinants devoid of the I-SceI site (Fig 1B).

### Verification of mutants

**Fig 2:**
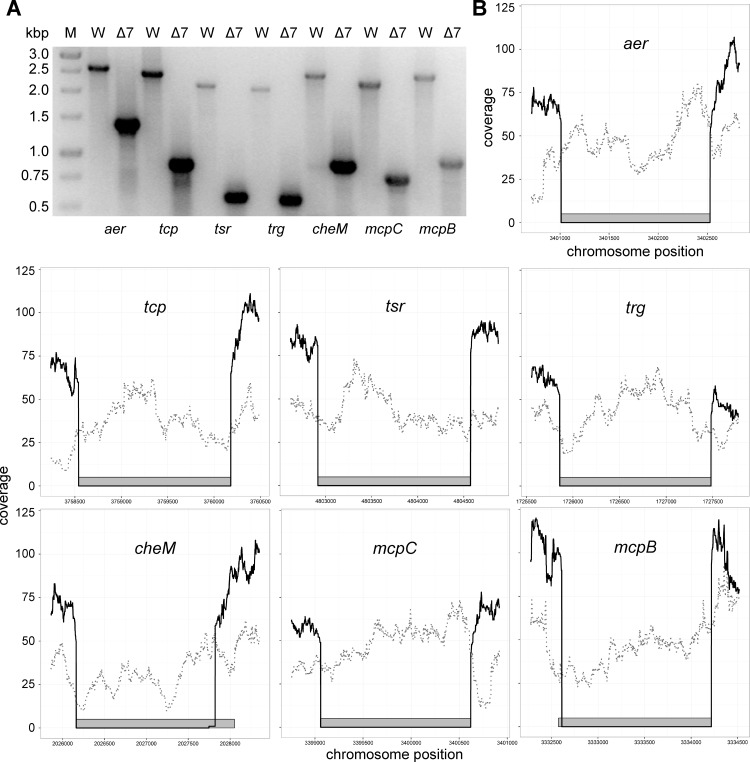
Genomic characterization of the WRG279 (Δ7 MCP) mutant. (A) Agarose gel showing PCR fragments generated using the primers as listed in Table 1 with either wild type (W) or WRG279 (Δ7) chromosomal DNA as a template. Shorter PCR products of the expected sizes confirmed gene deletion for the seven loci in WRG279. M = DNA marker, band sizes are shown in kbp (B) Next generation sequencing coverage data of strain WRG279 (black line) and NCTC 12023 WT (dotted gray line) is shown for the targeted MCP genes (gray rectangles) including 300 nucleotides before and after each coding sequence. For strain WRG279 no sequencing reads were obtained for the deleted genes and thus a lack of coverage was observed at the respective nucleotide positions of the wild type sequence.

Successful scarless deletion of each MCP gene was verified after the 2^nd^ recombination step by PCR using primers which bind up- and downstream of the site of deletion, respectively. Using these primer combinations, mutant alleles should result in shorter fragments compared to the WT situation. The theoretical fragment lengths for mutant and WT of each MCP gene are listed in Table 1. Starting from the single deletion strain WRG246 Δ*aer* all scarless mutants were checked whether their MCP alleles corresponded to the expected genotype. Agarose gels that summarize the individual allele types for each of the MCP genes of the seven sequential deletion strains are depicted in S1 Fig. No PCR fragments were observed for the ‘*aer*’ locus in the Δ6 (WRG277) and Δ7 (WRG279) strains. Since the *mcpC* gene is located adjacent to *aer*, deletion of *mcpC* removed the reverse primer binding site in these mutants. All other observed fragments were of the expected size as listed in Table 1. Fig 2A shows a direct comparison of the PCR products originating from WT genomic DNA or from WRG279 lacking all seven MCP genes (Δ7) which confirmed the absence of the MCP genes in the Δ7 strain. Finally, Sanger sequencing of the PCR fragments verified the expected nucleotide sequence of all MCP deletion sites (data not shown).

**Table 1:** Expected fragment sizes of verification PCRs.

Locus	Forward primer	Reverse primer	Fragment WT [bp]	Fragment after deletion [bp]
*aer*	Aer-Delcheck-for2	McpC-Delcheck-rev	2605	1370
*tcp*	Tcp-Delcheck-for	Tcp-Delcheck-rev	2150	509
*tsr*	Tsr-Delcheck-for	Tsr-Delcheck-rev	2203	544
*trg*	Trg-Delcheck-for	Trg-Delcheck-rev	2153	530
*cheM* (*tar*)	CheM-Delcheck-for	CheM-Delcheck-rev	2555	896
*mcpC*	McpC-Delcheck-for	McpC-Delcheck-rev	2330	768
*mcpB*	McpB-Delcheck-for2	McpB-Delcheck-rev	2414	813

Expression of highly efficient phage recombinases such as λ Red can cause unwanted recombination events within the chromosome [40]. This is especially evident if “scars” e.g. from recombinases such as Flp or Cre accumulate in the genome during multi-step mutagenesis protocols. It has been shown that a set of mutated recombination sites provides one possible but laborious solution to circumvent this problem [41]. There is no need for such amendments using a scarless protocol as presented in this study. Given the mutagenic activity of extended high-level expression of λ Red recombinase [40], it is very important to strictly limit λ Red expression when multiple successive mutation steps are carried out in the same bacterial background. With the pSIM5-based Red expression plasmid pWRG730 10–15 minutes of heat induction is sufficient for efficient recombination [42].

Despite all these precautions we could not completely exclude accumulation of mutations in the strain WRG279. To address this, Illumina-based whole genome sequencing of our NCTC 12023 WT laboratory strain and of the Δ7 MCP mutant WRG279 was carried out. The obtained sequencing reads were mapped to the published genome sequence of STM ATCC 14028S which is isogenic with strain NCTC 12023. In contrast to NCTC 12023 WT, no sequencing reads of WRG279 were mapped to the deleted MCP genes (Fig 2B). Furthermore, the coverage data of WRG279 confirmed the precise deletion sites as determined by the homology regions of the 2^nd^ TC (not shown). The ATCC 14028S genome data was also used as a reference to identify single nucleotide polymorphisms (SNPs). From the total 23 SNPs identified comparing ATCC 14028S with NCTC 12023 and WRG279 (data not shown) only two were unique for WRG279 (Table 2). One SNP resulted in a silent substitution within STM14_2710 and was detectable in all MCP mutants (Table 2). The other SNP was located within the putative ribosome binding site (RBS) of the *yceB* gene (STM14_1333) and appeared only in the last three mutants generated (Table 2). YceB is a predicted outer membrane lipoprotein and found to be downregulated in a resistant *Salmonella* strain isolated after a single challenge with nalidixic acid. Thus, this isolate exhibited additional resistance to tetracycline and chloramphenicol [43]. In contrast the WRG279 mutant showed minimal inhibitory concentrations (MICs) for both antibiotics similar to the parental NCTC 12023 WT strain in broth dilution assays (S2 Fig). However, modulation of YceB activity beyond antibiotic resistance due to altered protein levels cannot be excluded. These results argue against the *yceB* RBS mutation as an adaptation to the prolonged exposure to chloramphenicol, AHT, or both. Furthermore, both identified mutations are at chromosomal loci distantly located from the sites of recombination which makes their emergence unlikely to be directly linked to the recombination procedures applied. These low impact mutations might rather reflect natural genomic plasticity from prolonged laboratory handling as shown recently for chronic *Salmonella* infections [44, 45].

**Table 2:** Distribution of single nucleotide polymorphisms unique for WRG279.

Strain	Position* 1,208,402 RBS of *yceB* (STM14_1333)	Position* 2,345,688 synonymous mutation within STM14_2710
NCTC 12023 WT	A	G
WRG246	A	A
WRG255	A	A
WRG260	A	A
WRG264	A	A
WRG269	G	A
WRG277	G	A
WRG279	G	A

* reference: ATCC 14028S genome.

In summary our data highlight that the optimized system based on the dual-functional pWRG730 in combination with the pWRG717-derived kanamycin resistance cassette allows for fast and reliable manipulation of the *Salmonella* genome. This highly efficient tool is likely applicable in other bacteria amenable for the Red recombinase system, for example *E*. *coli* [36], *Shigella* spp. [46], *Yersinia enterocolitica* [47], *Y*. *pestis* [9], *Pseudomonas aeruginosa* [7] or *Pantoea ananatis* [48].

### Functional characterization of the MCP deletion strains

**Fig 3:**
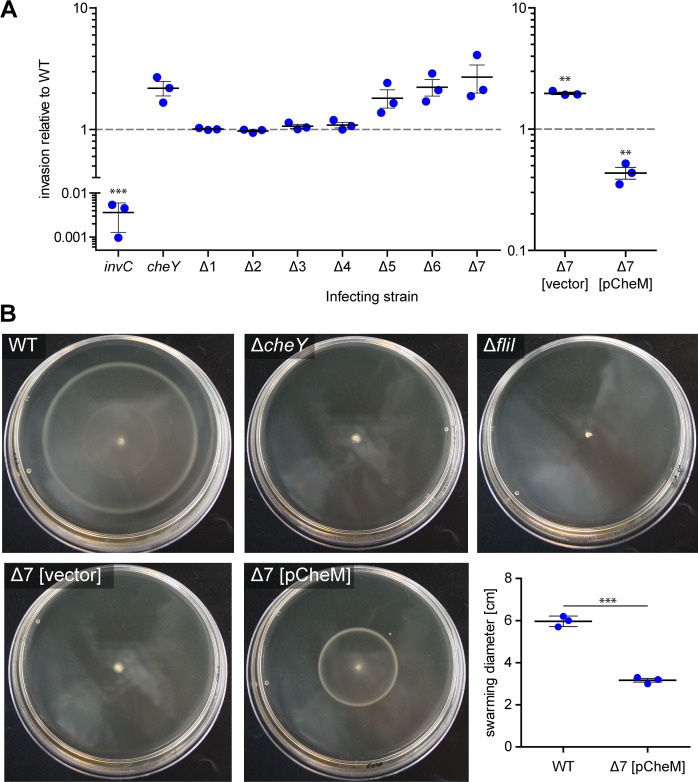
Functional characterization of the WRG279 mutant. (A) HeLa cells were infected with different STM strains and relative invasion rates compared to STM WT were calculated after one hour of infection. An *invC* mutant lacking a functional T3SS-1 was used as a negative control for invasion and a motile but non-chemotactic *cheY* mutant was included to evaluate the impact of directed motility. The Δ1 to Δ7 strains represent sequential MCP deletions as follows: Δ1 = WRG246 Δ*aer*; Δ2 = WRG255 Δ*aer*, Δ*tcp*; Δ3 = WRG260 Δ*aer*, Δ*tcp*, Δ*tsr*; Δ4 = WRG264 Δ*aer*, Δ*tcp*, Δ*tsr*, Δ*trg*; Δ5 = WRG269 Δ*aer*, Δ*tcp*, Δ*tsr*, Δ*trg*, Δ*cheM*; Δ6 = WRG277 Δ*aer*, Δ*tcp*, Δ*tsr*, Δ*trg*, Δ*cheM*, Δ*mcpC*; Δ7 = WRG279 Δ*aer*, Δ*tcp*, Δ*tsr*, Δ*trg*, Δ*cheM*, Δ*mcpC*, Δ*mcpB*. The right panel shows the invasion rates of the Δ7 strain complemented with pCheM (pWRG847) or transformed with the empty vector pWSK29 (vector). Statistical significance was calculated using a one sample *t* test against the hypothetical value 1.0 and was defined as ** for *p* < 0.01 and *** for *p* < 0.001. (B) Swarming phenotypes of different *Salmonella* strains as indicated on LB soft agar plates. Depicted is one representative out of three similar experiments. The diagram in the lower right panel shows the diameter of the swarming rings of *S*. Typhimurium WT and the Δ7 MCP mutant complemented with a CheM expression plasmid or a vector control as described in (B). Data of three independent biological replicates including means and standard deviations are shown. Statistical significance was calculated using a two-tailed paired Student’s *t* test and was defined as *** for *p* < 0.001.

Having confirmed the genetic integrity of the mutants we went on to functionally characterize the strains. It has been demonstrated previously that bacterial motility is an important factor for efficient invasion of host cells [49, 50]. Actively swimming bacteria encounter shear forces which bring them within close proximity of the host cell surface. This “near surface swimming” promotes *Salmonella*-cell interactions and cooperative invasion of membrane ruffles [51]. By utilizing a *cheY* mutant which uncouples motility from chemotaxis [26] it was demonstrated that directed swimming is not required for near surface swimming [51]. However, *in vivo* results using streptomycin-pretreated mice underlined the importance of chemotaxis as a major virulence function besides motility [52]. We set out to test whether the lack of multiple MCPs influences *Salmonella* invasion in a HeLa-based infection model. Quantification of intracellular bacteria was done after one hour and was normalized to STM WT (set to 1). Very low amounts of intracellular bacteria were detected for an *invC* deletion mutant harboring a non-functional SPI-1 encoded type three secretion system (T3SS-1) (Fig 3A, left panel). Our data revealed an approximately 2-fold increased invasion rate for the *cheY* mutant suggesting an inhibitory effect of chemotaxis on HeLa invasion (Fig 3A, left panel). This result is in line with previous observations where “smooth” swimming mutants such as *cheY* or *cheA* exhibited increased invasion capabilities in HEp-2 cells [49]. In contrast a “tumbling only” *cheB* mutant was shown to have lower tissue culture invasion rates [49] presumably due to decreased near surface swimming. Next we used the set of successive MCP deletion mutants to elucidate the impact of specific chemotactic signaling on HeLa cell invasion. Surprisingly, we found a clear phenotypical separation of two groups of mutants. The first group comprising mutants Δ1 (WRG246 Δ*aer*) to Δ4 (WRG264 Δ*aer*, Δ*tcp*, Δ*tsr*, Δ*trg*) exhibited invasion rates very similar to WT whereas the remaining mutants, which lack five to seven MCP genes, showed an elevated invasion comparable to a *cheY* mutant (Fig 3A, left panel).

Deletion of the *cheM* gene is the common feature of mutants Δ5 (WRG269) to Δ7 (WRG279). The observed phenotypes suggested that only CheM (Tar) is responsible for chemotactic signaling in the HeLa infection model supported by previous results obtained with HEp-2 cells [49]. To investigate this effect in more detail we complemented the Δ7 MCP strain WRG279 with the low-copy number plasmid pCheM (pWRG847) harboring the *cheM* gene under control of its natural promoter. While WRG279 transformed with the empty vector pWSK29 exhibited an elevated invasion similar to the *cheY* mutant, invasion of WRG279 [pCheM] was significantly decreased compared to WT (Fig 3A, right panel). We thus hypothesize that the six to eight plasmid copies per cell [53] led to an increased expression level of *cheM* which in turn fostered CheM-dependent chemotactic signaling with higher tumbling rates. CheM can directly sense aspartate [29]. Aspartate is not a component of the DMEM medium but small amounts of different amino acids are present in the FCS used during infection. The cell- and receptor density-dependent signaling preference of Tar-Tsr receptor complexes for either aspartate or serine could explain the apparent lack of chemotactic response from Tsr in our assays [54]. In the HeLa infection model aspartate or other so far uncharacterized CheM ligands might be liberated from the host cells thereby triggering a chemotactic response of *Salmonella*.

In a further set of experiments we wanted to characterize the MCP mutant strains in swarming assays on soft agar plates. As expected we observed a decrease in swarming diameter with more MCP genes deleted which reflects their increasing incapacity to perceive chemotactic signals (S3 Fig). Interestingly, the mutant WRG269 expressing only the MCPs McpB and McpC did show residual chemotactic movement whereas the successive WRG277, with *mcpC* deleted, was incapable of directed motility (S3 Fig). Like for WRG277, no chemotactic swarming could be observed for mutants lacking *cheY* or for the non-motile *fliI* mutant (Fig 3B). The Δ7 mutant WRG279 harboring the empty vector pWSK29 was phenotypically indistinguishable from the *cheY* mutant. Because both mutants are still motile they exhibited a slightly “blurred” inoculation site, which is most likely the result of “tumbly” swimming (Fig 3B). Introduction of the CheM complementation plasmid pCheM in WRG279 partially restored its chemotactic capacity (Fig 3B, lower right panel). Intriguingly, a single swarming ring could be detected for the CheM-complemented Δ7 MCP mutant which diameter roughly corresponded to the inner (CheM/Tar) ring observed for STM WT (Fig 3B). For STM WT two concentric rings were observed in some experiments which expanded over time to the agar plate periphery (Fig 3B). It has been demonstrated for *E*. *coli* that the outer ring corresponds to cells sensing serine through Tsr and the inner ring is composed of bacteria which sense aspartate by Tar [27, 28].

Our results and those of others [49] suggest that functional chemotaxis alone is disadvantageous for *Salmonella* invasion of HeLa and HEp-2 cells *in vitro*. However, the *in vivo* environment might be far more complex with chemotaxis being an important virulence factor [52, 55]. The optimized mutagenesis protocol described in the present study enabled us to efficiently generate a set of mutants lacking up to seven MCP genes. Further functional characterization of this set of MCP mutants *in vitro* identified CheM/Tar as the only MCP responding to chemotactic signals in a HeLa-based infection model. Future *in vivo* testing of these and other mutants, successfully generated by the developed procedure, might help to decipher the environmental signals *Salmonella* responds to during natural infection.

## Materials and methods

### Bacterial strains and plasmids

All strains used are listed in Table 3. Bacteria were routinely grown in LB media supplemented with 50 μg/mL carbenicillin (Cb) (Carl Roth, Mannheim, Germany), 25 μg/mL kanamycin (Km) (Carl Roth), 10 μg/mL chloramphenicol (Cm) (Carl Roth), 50 μg/mL spectinomycin (Sp) (Carl Roth) or 100 ng/mL anhydrotetracycline (AHT) (# 37919 Sigma-Aldrich, Schnelldorf, Germany) if required. Table 4 gives an overview of all the plasmids used in this study.

**Table 3:** Strains used in this study.

Strain	Relevant characteristic(s)	Source or Reference
MvP818	NCTC 12023 Δ*invC* FRT	[56]
MvP1212	NCTC 12023 Δ*cheY* FRT	[57]
MvP1213	NCTC 12023 Δ*fliI* FRT	[58]
NCTC 12023	Wild type, Nal^s^, isogenic to ATCC 14028	NCTC, Colindale, UK
WRG244	NCTC 12023 Δ*aer*::I-SceI *aph*, Km^r^	This study
WRG246	NCTC 12023 Δ*aer*	This study
WRG247	NCTC 12023 Δ*aer* Δ*tcp*::I-SceI *aph*, Km^r^	This study
WRG255	NCTC 12023 Δ*aer* Δ*tcp*	This study
WRG259	NCTC 12023 Δ*aer* Δ*tcp* Δ*tsr*::I-SceI *aph*, Km^r^	This study
WRG260	NCTC 12023 Δ*aer* Δ*tcp* Δ*tsr*	This study
WRG263	NCTC 12023 Δ*aer* Δ*tcp* Δ*tsr* Δ*trg*::I-SceI *aph*, Km^r^	This study
WRG264	NCTC 12023 Δ*aer* Δ*tcp* Δ*tsr* Δ*trg*	This study
WRG266	NCTC 12023 Δ*aer* Δ*tcp* Δ*tsr* Δ*trg* Δ*cheM*::I-SceI *aph*, Km^r^	This study
WRG269	NCTC 12023 Δ*aer* Δ*tcp* Δ*tsr* Δ*trg* Δ*cheM*	This study
WRG276	NCTC 12023 Δ*aer* Δ*tcp* Δ*tsr* Δ*trg* Δ*cheM* Δ*mcpC*::I-SceI *aph*, Km^r^	This study
WRG277	NCTC 12023 Δ*aer* Δ*tcp* Δ*tsr* Δ*trg* Δ*cheM* Δ*mcpC*	This study
WRG278	NCTC 12023 Δ*aer* Δ*tcp* Δ*tsr* Δ*trg* Δ*cheM* Δ*mcpC* Δ*mcpB*::I-SceI *aph*, Km^r^	This study
WRG279	NCTC 12023 Δ*aer* Δ*tcp* Δ*tsr* Δ*trg* Δ*cheM* Δ*mcpC* Δ*mcpB*	This study

**Table 4:** Plasmids used in this study.

Plasmid	Relevant characteristic(s)	Source or Reference
pDL1098	Temperature-sensitive mTn*10* delivery vector, Cm^r^, Sp^r^	[39]
pKD4	*aph* resistance cassette flanked by FRT sites, λ Pir dependent replication, Km^r^, Ap^r^	[36]
pSIM5	temperature-sensitive replication (30°C) and Red recombinase expression (42°C), Cm^r^	[38]
pWRG99	pKD46 [36] derivative, temperature-sensitive replication (30°C), arabinose-inducible expression of Red recombinase, Tet-inducible expression of I-SceI, Ap^r^	[4]
pWRG717	pBluescript II SK+ derivative, *aph* resistance cassette and I-SceI cleavage site, Km^r^, Ap^r^	This study
pWRG730	pSIM5 [38] derivative, temperature-sensitive replication (30°C) and Red recombinase expression (42°C), Tet-inducible expression of I-SceI, Cm^r^	This study
pWRG829	pBluescript II SK+ derivative, *aad9* resistance cassette and I-SceI cleavage site, Sp^r^, Ap^r^	This study
pWRG832	pWRG717 derivative, *aph* resistance cassette reversed, Km^r^, Ap^r^	This study
pWRG841	P_*cheM*_::*cheM* in pCRII-TOPO, Km^r^, Ap^r^	This study
pWRG847	pCheM; P_*cheM*_::*cheM* in pWSK29, Ap^r^	This study
pWRG865	pWRG829 derivative, *aad9* resistance cassette reversed, Sp^r^, Ap^r^	This study
pWSK29	Low-copy-number vector, Ap^r^	[53]

### PCR and cloning

All primers used for cloning are listed in S1 Table. For construction of the I-SceI *aph* template plasmid pWRG717, an I-SceI cleavage site was fused to the kanamycin resistance cassette of pKD4 by PCR using primers XhoI-aph-for2 and Aph-I-SceI-KnpI-rev2. The PCR fragment was cloned via XhoI/KpnI in pBluescript II SK+ (Agilent Technologies, Waldbronn, Germany). For construction of all other plasmids including the spectinomycin template plasmid pWRG829, assembly cloning of PCR fragments was used [59]. Primers pWSK29-Gbs-for and pKD4-Gbs-rev were used with pWRG717 as template to obtain a PCR fragment containing the vector and the I-SceI cleavage site. A spectinomycin resistance cassette was amplified with primers pSK-aad9-Gbs-for and pKD-aad9-Gbs-rev from plasmid pDL1098 [39] and subsequently combined with the first PCR product to obtain pWRG829. In template plasmids pWRG832 and pWRG865 the antibiotic resistance cassettes are in reversed orientation compared to pWRG717 and pWRG829, respectively. Here, pBluescript II SK+ was amplified with primers pWSK29-Gbs-for and -rev. The resistance cassettes were amplified from pWRG717 and pWRG829 with primer pairs Aph-I-SceI-pSK-Gbs-for/Aph-pSK-Gbs-rev2 and Aad9-I-SceI-pSK-Gbs-for/Aad9-pSK-Gbs-rev, respectively. A two-fragment assembly of each of the resistance cassettes with the vector PCR fragment led to the final plasmids. The heat-inducible Red recombinase expression plasmid pSIM5 [38] was linearized by PCR using primers pSIM-Gbs-for and pSIM-Gbs-rev. The tetracycline-inducible I-SceI expression cassette was amplified with primers pSIM-TetR-Gbs-for2 and pSIM-I-SceI-Gbs-rev using pWRG99 [4] as template. The two PCR fragments were combined by assembly cloning resulting in plasmid pWRG730. The region containing the *cheM* promoter and coding sequence was amplified from chromosomal DNA using primers CheM-Delcheck-for and CheM-pWSK-Gbs-rev. The PCR fragment was blunt-cloned in pCR II-TOPO (Thermo Fisher Scientific, Karlsruhe, Germany) resulting in plasmid pWRG841 which was subsequently digested with KpnI and XbaI. The *cheM*-containing fragment was gel-purified and cloned in the similarly-digested pWSK29 to obtain pWRG847 (pCheM).

### Generation of mutants

All primers used to amplify the kanamycin resistance cassette from pWRG717 and to obtain a TC from *Salmonella* genomic DNA are listed in S1 Table. The desalted primers were purchased from Integrated DNA Technologies (Munich, Germany). For amplification of the first targeting construct forward primers consisting of the 3’ sequence 5’-AGGGTTTTCCCAGTCACGAC-3’, which binds to all pBluescript II SK+ -based template vectors, and a 5’ 40 bases sequence homologous to the genomic target site were used. The 60-mer reverse primers were similarly designed with the following 3’-located sequence binding to the template vectors: 5’-TGCTTCCGGCTCGTATGTTG-3’. Overnight (O/N) cultures of *Salmonella* harboring pWRG730 were grown at 30°C in LB supplemented with 10 μg/mL chloramphenicol. O/N cultures were re-inoculated 1:100 in fresh medium and grown aerated to an OD_600_ of 0.3 to 0.5. Red recombinase expression was induced for 12.5 minutes in a shaking water bath at 42°C [42]. After that bacteria were immediately put on ice and electro-competent cells were prepared essentially as described before [4]. Cells were transformed with 100–500 ng purified 1^st^ TC using a Micropulser device (Bio-Rad, Munich, Germany) at ‘EC2’ setting. Successful recombinants were selected on LB plates containing chloramphenicol (plasmid pWRG730) and kanamycin (*aph* cassette from pWRG717 template) and kept at 30°C to preserve pWRG730. Colony-PCRs with suitable primers were routinely used to check for correct insertion of the resistance cassette within the genome in both directions. For subsequent removal of the resistance cassette competent cells were prepared from confirmed mutants still harbouring pWRG730 as described above. After transformation a 10-fold dilution series of the cells up to 10^−4^ was prepared in LB and plated on LB agar containing chloramphenicol and AHT. Plates were kept O/N at 30°C and large colonies were picked and purified again on LB agar plates containing chloramphenicol and AHT. Successful deletion of a MCP gene was confirmed with PCR using primers binding to the flanking regions of the gene (see Table 1) and subsequent Sanger sequencing of the PCR products (data not shown). After going through the desired number of deletion cycles, plasmid pWRG730 was cured from the bacteria by O/N incubation at 42°C.

### Whole genome sequencing and mapping

Genomic DNA of strain NCTC 12023 wild type (WT) and the isogenic 7x MCP mutant WRG279 was prepared from O/N cultures in LB medium using a GenElute Bacterial Genomic DNA kit (Sigma-Aldrich, Schnelldorf, Germany) according to manufacturer’s instructions. One ng of genomic DNA of each strain was fragmented using the Nextera sample preparation kit (Illumina, San Diego, CA, USA) and sequenced on a MiSeq (Illumina) machine running in paired end mode with 300 bp read length. All raw sequence reads of BioProject PRJNA355390 (http://www.ncbi.nlm.nih.gov/bioproject/) are available through SRA (http://www.ncbi.nlm.nih.gov/sra) accessions SRR5062192 (WT) and SRR5062193 (WRG279).

Consensus sequences for the two genomes were determined utilizing a custom in-house analysis pipeline as described earlier [60]. Briefly, MiSeq reads were mapped to the published genome sequence of *S*. Typhimurium strain ATCC 14028S (accession: CP001363) by a combination of BWA-SW version 0.7.13-r1126 [61] and SAMtools 0.1.19 [62]. VarScan 2.3 [63] was utilized for consensus calling. After mapping sequencing coverage was extracted using SAMtools 1.3.1 and visualized with ‘ggplot2’ [64] from ‘R’ 3.3.0 [65]. SNPs were extracted from the NCTC 12023 WT and WRG279 genomes obtained by reference-based mapping using a custom in-house Python script. Regions containing SNPs unique for WRG279 or sites of MCP deletion were PCR-amplified and subjected to Sanger sequencing (GATC Biotech, Cologne, Germany) using suitable primers listed in S1 Table. Sequence data is available through BioProject PRJNA355390.

### Minimal inhibitory concentration assay

O/N cultures of test strains were diluted 1:100 in fresh LB and grown at 37°C to an OD_600_ between 0.5 and 0.7. After adjusting the cultures to an OD_600_ of 0.0002 (approximately 2 × 10^5^ bacteria/mL) in fresh 2-fold concentrated LB, 100 μl were added to each well of a 96-well plate containing 100 μl of increasing concentrations of chloramphenicol or tetracycline in distilled water. The plates were incubated at 37°C in a humid chamber for 16 h and absorbance was measured at 600 nm (Tecan Infinite M1000). The MICs were calculated using ‘R’ as described before [66].

### Cell culture and infection

HeLa cells (LGC Standards, Wesel, Germany) were grown in DMEM (Biowest, Germany) supplemented with 10% FCS, sodium pyruvate and 2 mM GlutaMax (Thermo Fisher Scientific, Karlsruhe, Germany) under humidified atmosphere with 5% CO_2_. Gentamicin protection assays were essentially carried out as described previously [67]. Briefly, 5 × 10^4^ HeLa per well were seeded in 24-well plates (Cell-star, Greiner bio-one, Frickenhausen, Germany) 24 h prior infection. Bacterial O/N cultures grown in LB supplemented with appropriate antibiotics were reinoculated 1:31 in fresh medium and grown aerobically for another 3.5 h. An inoculum corresponding to a multiplicity of infection (MOI) of 10 was prepared in DMEM and used to infect the HeLa cells for 25 min. After the cells were washed thrice with PBS, 500 μl of DMEM containing 100 μg/mL gentamicin was applied to each well to kill remaining extracellular bacteria. After one hour of incubation the cell layers were washed again with PBS and then lysed for 10 min with PBS containing 1% Elugent (Merck Millipore, Darmstadt, Germany) and 0,0625% Antifoam B (Sigma-Aldrich, Schnelldorf, Germany) to liberate the intracellular bacteria. Serial dilutions of the inoculum and the lysates were plated on Mueller Hinton (MH) plates to determine the colony-forming units. Based on the inoculum the percentage of invasive bacteria was calculated and subsequently normalized to WT.

### Swarming assay

Swarming of different *Salmonella* strains was assessed on LB semi-solid agar plates (LB with 5 g/L NaCl, 0.5% agar). A small amount (0.2 μl) of bacterial O/N cultures was applied onto the center of LB soft agar plate and incubated for six hours at 37°C. The diameters of the swarm colonies were measured and the plates were photographed.

## Supporting information

S1 Fig PCR fragments after colony PCR of sequential MCP deletion strains. Primer combinations as listed in Table 1 were used. One exception was the ‘*aer*’ locus where primer ‘Aer-Delcheck-rev2’ instead of ‘McpC-Delcheck-rev’ was used producing a 1087 bp fragment. Due to deletion of the reverse primer binding site during replacement of *mcpC* and *mcpB*, no product was observed (*) for WRG277 (6) and WRG279 (7). 1 = WRG246 Δ*aer*; 2 = WRG255 Δ*aer*, Δ*tcp*; 3 = WRG260 Δ*aer*, Δ*tcp*, Δ*tsr*; 4 = WRG264 Δ*aer*, Δ*tcp*, Δ*tsr*, Δ*trg*; 5 = WRG269 Δ*aer*, Δ*tcp*, Δ*tsr*, Δ*trg*, Δ*cheM*; 6 = WRG277 Δ*aer*, Δ*tcp*, Δ*tsr*, Δ*trg*, Δ*cheM*, Δ*mcpC*; 7 = WRG279 Δ*aer*, Δ*tcp*, Δ*tsr*, Δ*trg*, Δ*cheM*, Δ*mcpC*, Δ*mcpB*, M = DNA marker, band sizes indicated in kbp.
(TIF)

S2 Fig Determination of the minimal inhibitory concentration (MIC). MICs for chloramphenicol (left) and tetracycline (right) were determined for either *S*. Typhimurium NCTC 12023 wild type (WT) or the isogenic mutant WRG279 lacking seven MCP genes in broth dilution assays. Data of three independent biological replicates done in duplicates with means and standard deviations are shown. n.s. = not significant as calculated using a two-tailed unpaired Student’s *t* test.
(EPS)

S3 Fig Swarming phenotypes of different MCP mutants. (A) Swarming phenotypes on LB soft agar plates of different *Salmonella* MCP mutants as indicated. Depicted is one representative out of three similar experiments. (B) Diameters of the swarming rings with means and standard deviations of the different *Salmonella* strains from (A) for three independent biological replicates are shown. Statistical significance compared to WT was calculated using a two-tailed paired Student’s *t* test and was defined as * for *p* < 0.05 and *** for *p* < 0.001.
(TIF)

S1 Table Oligonucleotides used in this study. (XLSX)
