# Supplementary figures and images for: Scarless deletion of up to seven methyl-accepting chemotaxis genes with an optimized method highlights key function of CheM in Salmonella Typhimurium
Source: PLoS One. 2017 Feb 17;12(2):e0172630. doi: 10.1371/journal.pone.0172630 (PMC5315404; doi:10.1371/journal.pone.0172630)

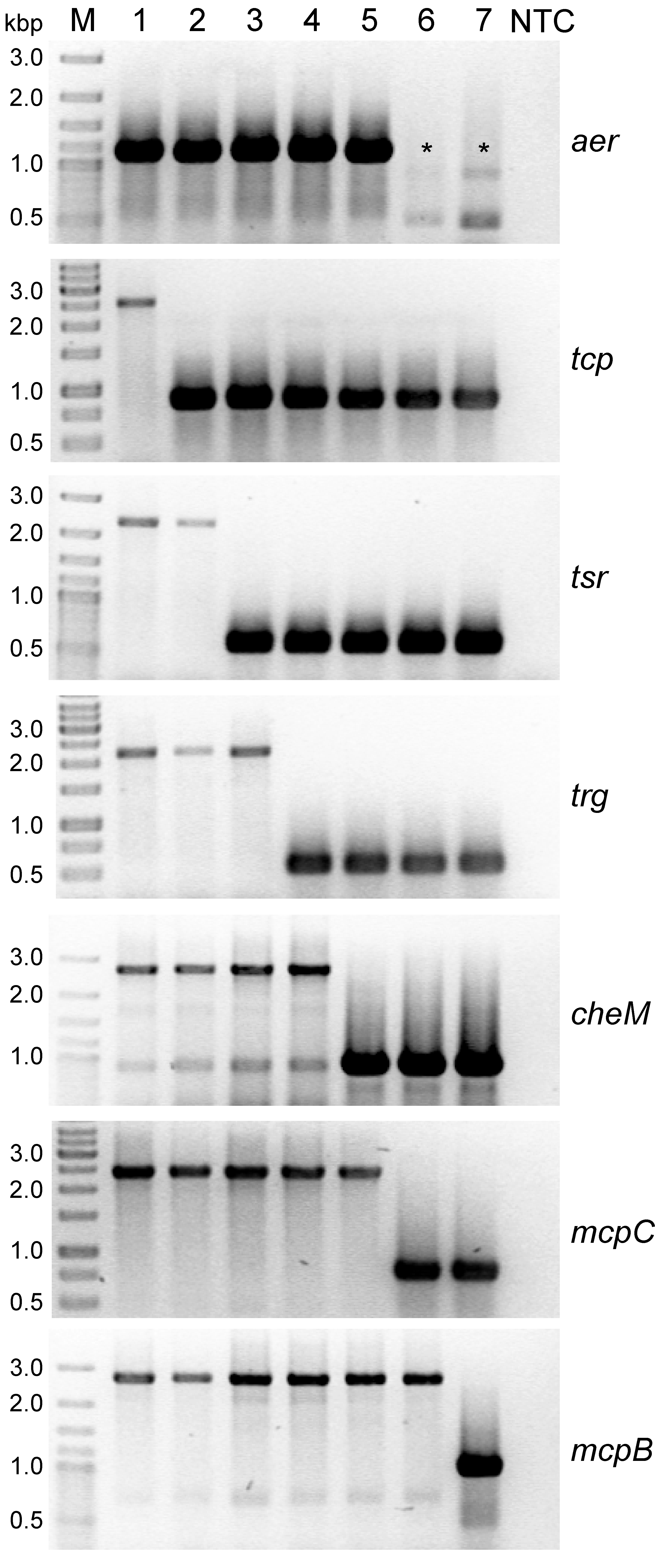

Supplement: S1 Fig — Primer combinations as listed in Table 1 were used. One exception was the ‘aer’ locus where primer ‘Aer-Delcheck-rev2’ instead of ‘McpC-Delcheck-rev’ was used producing a 1087 bp fragment. Due to deletion of the reverse primer binding site during replacement of mcpC and mcpB, no product was observed (*) for WRG277 (6) and WRG279 (7). 1 = WRG246 Δaer; 2 = WRG255 Δaer, Δtcp; 3 = WRG260 Δaer, Δtcp, Δtsr; 4 = WRG264 Δaer, Δtcp, Δtsr, Δtrg; 5 = WRG269 Δaer, Δtcp, Δtsr, Δtrg, ΔcheM; 6 = WRG277 Δaer, Δtcp, Δtsr, Δtrg, ΔcheM, ΔmcpC; 7 = WRG279 Δaer, Δtcp, Δtsr, Δtrg, ΔcheM, ΔmcpC, ΔmcpB, M = DNA marker, band sizes indicated in kbp. (TIF) [file pone.0172630.s001.tif]

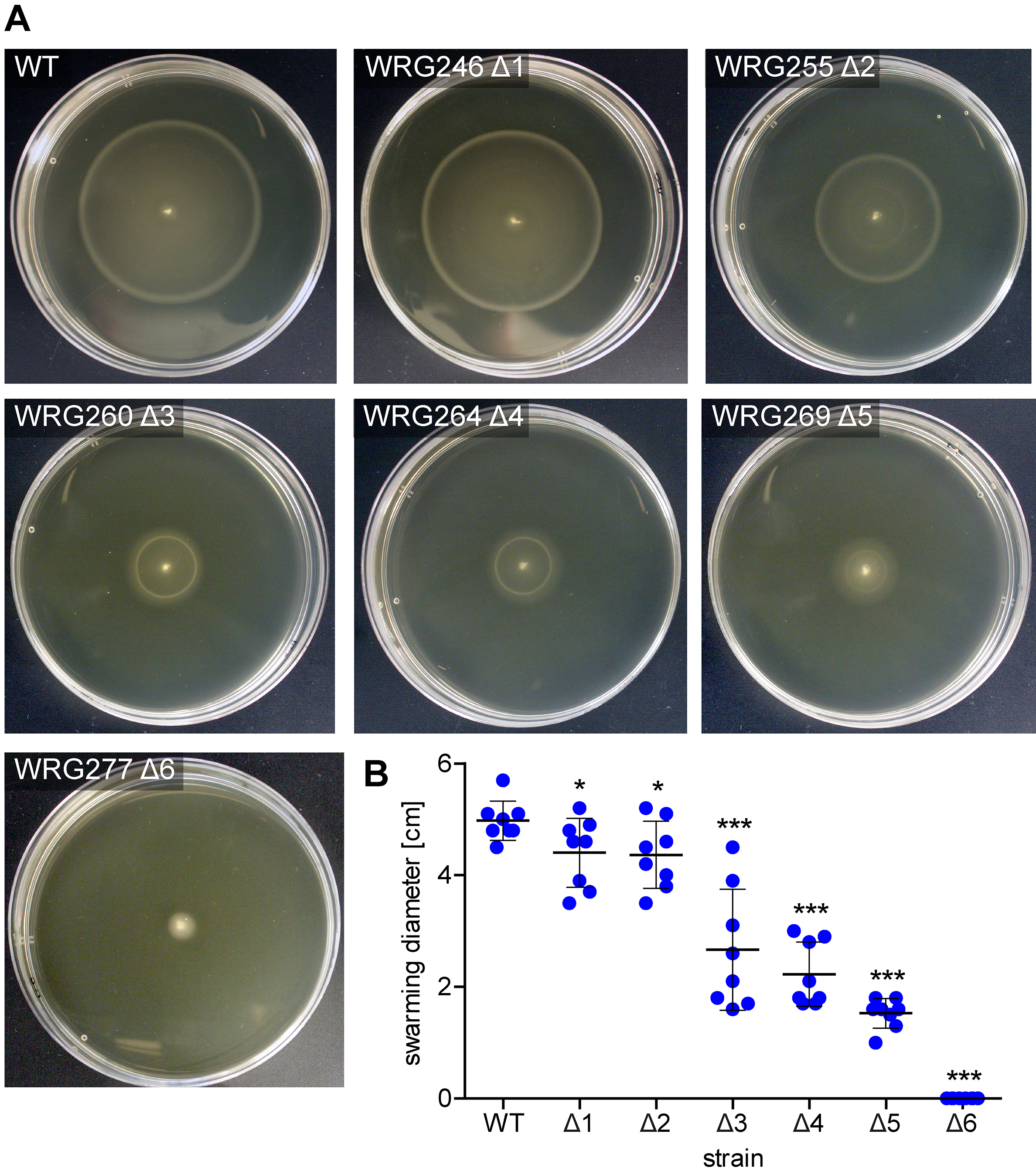

Supplement: S3 Fig — (A) Swarming phenotypes on LB soft agar plates of different Salmonella MCP mutants as indicated. Depicted is one representative out of three similar experiments. (B) Diameters of the swarming rings with means and standard deviations of the different Salmonella strains from (A) for three independent biological replicates are shown. Statistical significance compared to WT was calculated using a two-tailed paired Student’s t test and was defined as * for p < 0.05 and *** for p < 0.001. (TIF) [file pone.0172630.s003.tif]
